# Private costs almost equal health care costs when intervening in mild Alzheimer's: a cohort study alongside the DAISY trial

**DOI:** 10.1186/1472-6963-9-215

**Published:** 2009-11-25

**Authors:** Rikke Søgaard, Jan Sørensen, Frans B Waldorff, Ane Eckermann, Dorthe V Buss, Gunhild Waldemar

**Affiliations:** 1 CAST - Centre for Applied Health Services Research and Technology Assessment, University of Southern Denmark, Denmark; 2 The Memory Disorders Research Group, Department of Neurology, Rigshospitalet, Copenhagen University Hospital, Denmark; 3 Research Unit and Department of General Practice, Institute of Public Health, University of Copenhagen, Denmark

## Abstract

**Background:**

Alzheimer's disease is the leading cause of dementia and affects about 25 million people worldwide. Recent studies have evaluated the effect of early interventions for dementia, but few studies have considered private time and transportation costs associated with the intervention. This study assessed the total economic costs associated with a multifaceted intervention for mild Alzheimer's disease, including an estimate of the ratio of public to private costs.

**Methods:**

The study sample comprised 163 dyads of patients and caregivers who received a multifaceted intervention of counselling sessions, courses and informational packages. The typical duration of the intervention was 7 months. A micro-costing approach was applied using prospectively collected data on resource utilisation that included estimates of participant time and transportation. Precision estimates were calculated using a bootstrapping technique and structural uncertainty was assessed with sensitivity analysis.

**Results:**

The direct intervention cost was estimated at EUR 1,070 (95% CI 1,029;1,109). The total cost (including private costs) was estimated at EUR 2,020 (95% CI 1,929;2,106) i.e. the ratio of private to public costs was almost 1:1.

**Conclusion:**

Intervention for mild Alzheimer's disease can be undertaken at a relatively low cost to public funds. However, policy planners should pay attention to the significant private costs associated with an intervention, which may ultimately pose a threat to equity in access to health care.

**Trial registration:**

Current Controlled Trials ISRCTN74848736.

## Background

Recent estimates suggest that 24.3 million people worldwide have dementia and that every year 4.6 million new cases occur [1]. The prevalence of dementia has thus been significantly underestimated and its incidence will increase in the coming years due to demographic shift [2]. Alzheimer's disease (AD) is the leading cause of dementia, accounting for more than half of the cases [3].

The cost of care for people with dementia, sometimes divided into the cost of formal care and that of informal care, is related to the stage of disease. The UK combined annual 2005/6 costs for people with dementia living in the community were estimated at £16,689, £25,877 and £37,473 per person for mild, moderate and severe dementia, respectively [4]. US estimates are generally higher but, due to methodological differences, wide variation is demonstrated across studies [5,6]. None of these studies included valuation of reduced quality of life experienced by the person with dementia or the caregiver, i.e. cost estimates represent minimum values.

Dementia is a chronic condition and intervention typically aims at slowing down disease progress and improving quality of life for the person with dementia. However, dementia affects not only the patient but also the caregivers, who are often family members or others close to the patient [7]. The gradual loss of the patient's cognitive ability exerts a significant emotional strain on the caregiver, and this can have implications for the caregiver's social or working life [6,8]. From both clinical and health economic perspectives, therefore, it is important that interventions for dementia take into account effects on caregivers.

The rationale for early intervention in dementia is clear, but there is no apparent gold standard for the most appropriate approach. Current proposals include training and education programmes, information and technology-based support including specialised computer and telephone networks, and more formal approaches to planning care [9]. Some of these have been assessed for economic efficiency and found to be cost effective [10-13] but none have included the resource category of participant time, i.e. the time required from participants to attend therapeutic regimens. This need not be vital when providing decision-makers with a foundation for prioritizing resources under the objective of allocative efficiency but, when prioritizing resources under the additional objective of equity in access to care, the burden of private costs becomes highly relevant [14].

Even in a publicly financed health care system, users are faced with copayment due to transportation costs, out-of-pocket costs and the opportunity cost of their time spent receiving therapy. These costs, ceteris paribus, increase with the duration and intensity of a therapeutic regimen and are further increased if the regimen is targeted to multiple parties e.g. patients and caregivers. Costs to private parties associated with interventions for mild AD thus pose a challenge to health policy planning in that the implementation of new interventions may have detrimental effects on equity; less resourceful patients or caregivers may decline participation due to excessive private costs or, if accepting therapy, they may experience significant financial strain. This consequence has been neglected in the AD literature [15].

This paper assesses intervention costs of a best-bet-intervention in mild AD using a micro-costing approach to facilitate precise estimation of public and private costs. It should not be confused with the literature assessing the extent or interrelationship of formal and informal costs. This study takes a narrower view of the extent of copayment in a population that is particularly vulnerable due to the nature of the therapeutic regimen - which is often long term and requires time and input from caregivers. The aim of this study was to assess the total economic costs of a multifaceted intervention in mild AD, as well as the ratio of public to private costs.

## Methods

### Sample and study design

The study sample of 163 dyads of patients and their caregivers was selected from a multi-centre, randomised, controlled trial examining the efficacy of multifaceted intervention aimed at patients and their caregivers. The study sample included participants assigned to the intervention arm only, as the control group received no intervention. The trial was conducted across five (out of 15) Danish county districts. Inclusion criteria comprised age ≥ 50 years, a recent diagnosis of AD (within the past 12 months), Mini Mental State Examination (MMSE) score ≥ 20, and having a primary caregiver who was willing to participate in the study. The diagnosis of dementia was determined in consensus with fourth edition of the The Diagnostic and Statistical Manual of Mental Disorders [16] and the subtype of AD was determined using the NINCDS-ADRDA criteria [17]. Exclusion of patients with Lewy body disease was based on the McKeith criteria [18]. Furthermore, institutionalised patients and patients with severe comorbidity were excluded. The present study is thus a cohort study following persons with AD and their caregivers from the first contact they had as part of the intervention (initial meeting) to the last follow-up (final meeting), which typically took place six to twelve months after the initial meeting. The first study patients presented in January 2004 and the final follow-up visit was in June 2006.

### Intervention

A multifaceted intervention was designed with the objective of preventing or reducing depressive symptoms, impairments of health-related quality of life and loss of social network. The philosophy was to focus on positive resources, intact functions and retained skills, and activities that patients could still participate in. The following sections describe key components of the intervention.

### Counselling

A series of five counselling sessions, comprising both individual sessions and group participation, was scheduled. Counselling was based on constructivist principles [19] and supported by the use of written notes, that were established at the initial meeting and used to focus follow-up sessions, according to Ishiayma's ideas on self-validation [20]. A designated nurse undertook all counselling sessions after receiving specialised training in the constructivist approach. In addition to face-to-face time, the counsellor had an average preparation time of 30 minutes before an initial meeting and 10 minutes before a follow-up meeting. Participating study centres decided individually whether counselling was undertaken in the patient's home or at the centre; approximately one-third of the sessions took place in the patients' homes.

### Courses

Two parallel lines, each of five courses, were aimed at patients and caregivers, respectively. The objective was to provide basic knowledge about dementia and its consequences as well as to establish a forum for patients and caregivers to exchange experiences and coping strategies. Patient-targeted courses included written and oral information on key issues about dementia and its consequences. Caregiver-targeted courses included more formal education about AD, supplemented by written information. All courses were scheduled for 12 participants per session and were run by one counsellor and one invited teacher. Patient-targeted courses were additionally staffed by two volunteers, who assisted the professionals during the courses. Course duration was fixed at two hours (with an additional 30 minutes of preparation time for the counsellor).

### Telephone counselling

At inclusion into the study, patients and caregivers were informed that the intervention would include a regular telephone call from the counsellor (approximately five to eight times in intervals of three to four weeks) to ensure regular contact and to follow up on issues discussed in individual counselling sessions. Patients and caregivers were asked to decide who should receive the telephone calls; this could be either or both parties.

### Handbook and Logbook

A comprehensive handbook about dementia was composed for the study; one part was targeted patients and the other caregivers. The handbook contained chapters on causes of dementia, legal aspects in relation to living with dementia, and sources and contacts for social support. The handbook included a logbook section to encourage patients and caregivers to write about their daily lives and activities. However, since only six patients and seven caregivers did so, this information was not included in the present analysis.

### Concordance

The intervention comprised a range of components that participants could choose to take advantage of. Participation was thus a result of the counsellor's assessment of individual needs and preferences in collaboration with the participant's (patient, caregiver, network) wishes. The term 'compliance' was thus avoided and replaced by the term 'concordance' to emphasise that target participation rates were not necessarily 1.00. Concordance is typically defined as adherence to an agreed schedule rather than adherence to a fixed regimen.

### Costing

The intervention was costed from a societal perspective, including the costs of both formal and informal time of the persons involved, and based on concurrent data collection on resource utilisation.

The costs of developing or implementing the intervention were not included in the present analysis, which should be seen as estimating long-term average costs of implementing the intervention in routine practice. As the intervention was designed as a flexible framework from which a tailored course could be set for each dyad, the cost analysis was conducted as a per-protocol analysis with service utilisation counting only if it did in fact occur.

A case report form - administered by the counsellor - was used to register the time (minutes) spent by professional staff, the patient, the caregiver and the patient's network across the scheduled activities of the intervention: counselling, telephone counselling and courses. A load-factor of 1.56 was applied to the cost of counselling to account for non-productive time, i.e. effective confrontation time was assumed to amount to, on average, 45 minutes of an hour (load of 0.25) due to pauses, walking distance between locations, private time etc. The remainder load (0.31) was based on internal statistics on absence from work due to vacation, sickness etc.

Valuation of formal care was based on gross market wage rates from national collective agreements between Danish local government and the respective professional associations except for teaching activity at courses, which was valued using market prices. An overhead rate of 20% was applied to account for capital costs.

Valuation of informal time, i.e. time spent by patients, caregivers and networks participating in the intervention, was undertaken using the opportunity cost method, in which the value of a person's time is reflected by his or her wage rate. National average gender- and age-matched salaries were used; for valuing leisure time the net salary was used and for valuing productive time the gross salary was used (both available from http://statistikbanken.dk  [21]). Transportation costs in relation to counselling sessions in the patients' homes were applied as an average base cost for all sessions, representing 20 minutes of time and 10 kilometres each way. Similarly, participants' transportation costs were applied as an average base cost of 30 minutes of time and 15 kilometres each way for all contacts. The governmental tariff for transportation by private car was used and it was further assumed that patients, caregivers and/or network participants shared one car. Item costs not available in 2008 prices were inflated using the general price index. Table 1 lists the item costs used.

**Table 1:** Item costs used for estimating the costs of a support programme for mild Alzheimer's disease.

Resource	Cost	Source
Time costs of formal care		
Counsellor (specially trained nurse)	25.93	Local Government Denmark [KL]
Load-factor for counsellor's time [weight]^1^	1.56	Local Government Denmark [KL]/estimate
Course teacher, nurse [per course]	131.69	Market prices (tariff of trial management)
Course teacher, academic [per course]	219.48	Market prices (tariff of trial management)
Volunteers assisting course personnel	17.73	National average net salary for 50-year olds
Capital costs	20%	Local Government Denmark [KL]
Time costs of informal time (examples)^2^		
Male, 50-54 years, gross	32.50	Statistics Denmark
Male, 50-54 years, net	19.76	Statistics Denmark
Female, 50-54, gross	23.40	Statistics Denmark
Female, 50-54, net	15.70	Statistics Denmark
Transportation cost professionals [EUR/km]	0.25	Danish Government
Transportation cost participant [EUR/km]	0.47	Danish Government

^1^A load factor was applied to account for non-productive time of a counsellor due to pauses, walking distance between locations, private time, vacation and other absence from work etc. ^2^A complete set of age- and gender-specific national average salaries was used (complete data are publicly available from StatBank Denmark at http://statistikbanken.dk).

All costs are in 2008 EUR per hour unless otherwise stated.

### Statistical analysis

Baseline characteristics of patients and caregivers were described using conventional parametric statistics and simple frequencies with percentage proportions. All other reported estimates are arithmetic means with 95% bias-corrected, bootstrapped confidence intervals (95% CIs) from a non-parametric bootstrap distribution, which was estimated from 1,000 extract replications from the original sample (n = 163). The technique of bootstrapping is a resampling technique that is appropriate for estimating confidence intervals in the presence of e.g. positively skewed data [22]. Statistical analyses were conducted using STATA version 10.0 (StataCorp, Texas).

### Ethics

The trial was conducted in accordance with the Helsinki declaration and was approved by the local ethics committee. All patients and caregivers provided written informed consent.

## Results

### Baseline characteristics

A total of 163 dyads were included, but only 157 dyads received the intervention; three dyads dropped out due to the intervention being too demanding for caregivers, one dyad dropped out due to the intervention being too demanding for the patient, one dyad dropped out because the patient was hospitalised due to comorbidity, and one dyad dropped out because the patient died before the intervention started.

All patients were non-institutionalised persons with a mild form of AD, as indicated by a mean MMSE score of 24 (SD 2). Mean age was 76 (SD 8) years with an almost equal distribution between genders. Baseline characteristics are further detailed in Table 2. Caregivers were characterised by a mean age of 65 (SD 13) years, a gender distribution with 66% women and a health-related quality of life of 0.83 (SD 0.17), which is comparable to an age- and gender-matched population norm. Nearly 60% were pensioners and approximately one-third were employed. The caregivers were typically the patient's spouse or son/daughter (in-law).

**Table 2:** Baseline characteristics of the study population of patients with mild Alzheimer's disease and their primary caregiver.

	Patient (n = 157)	Caregiver (n = 157)
Age, mean (SD)	76 (8)	65 (13)
Females, n (%)	85 (54)	104 (66)
Living alone (%)	53 (34)	22 (14)
MMSE score, mean (SD)	24 (2)	NA
EQ-5D score (self-assessed), mean (SD)	0.85 (0.17)	0.83 (0.17)
Occupational status, n (%)		
On pension	148 (94)	92 (59)
On early retirement	4 (3)	10 (6)
Working	3 (2)	49 (31)
Other (e.g. housewife)	2 (1)	6 (4)
Caregiver's relation to patient, n (%)		
Spouse	NA	99 (63)
Son/daughter (in-law)	NA	48 (31)
Other	NA	10 (6)

MMSE = Mini Mental State Examination. NA = Not applicable. EQ-5D = EuroQol 5-Dimensions

### Concordance

The participation rates (Table 3) were unexpectedly high, given the intervention's long duration, the many scheduled contacts and the associated transportation times and costs. Thus 88% of patients and 91% of caregivers participated in at least three counselling sessions, while 81% of patients and 80% of caregivers participated in at least three courses. While only one session (the network meeting) was aimed directly at patients' networks, these people were welcome to attend the other meetings and sessions; this opportunity was rarely used, however.

**Table 3:** Rates of participation (95% confidence intervals) in a flexible, multifaceted intervention for mild Alzheimer's disease.

	Patient (n = 157)	Caregiver (n = 157)	Network (n = 157)
Constructivist counselling			
Initial meeting	1.00	0.99 (0.98;1.00)	0.05 (0.02;0.09)
1^st ^follow-up meeting	0.84 (0.78;0.90)	0.85 (0.79;0.92)	0.05 (0.02;0.09)
2^nd ^follow-up meeting	0.78 (0.72;0.85)	0.78 (0.72;0.85)	0.05 (0.02;0.09)
Network meeting	0.32 (0.25;0.39)	0.32 (0.25;0.40)	0.29 (0.22;0.36)
Final meeting	0.83 (0.78;0.89)	0.82 (0.76;0.87)	0.09 (0.04;0.13)
Telephone counselling^2^	0.36 (0.28;0.43)	0.80 (0.73;0.86)	Not applicable
Courses			
1^st ^session	0.84 (0.78;0.90)	0.83 (0.77;0.89)	0.24 (0.17;0.30)
2^nd ^session	0.83 (0.77;0.89)	0.82 (0.76;0.88)	0.25 (0.19;0.32)
3^rd ^session	0.76 (0.70;0.83)	0.77 (0.70;0.84)	0.25 (0.18;0.32)
4^th ^session	0.76 (0.70;0.83)	0.75 (0.68;0.82)	0.17 (0.10;0.23)
5^th ^session	0.76 (0.70;0.83)	0.74 (0.66;0.80)	0.18 (0.12;0.25)
Participated in at least 3 counsellings^3^	0.88 (0.83;0.93)	0.91 (0.87;0.96)	0.05 (0.01;0.09)
Participated in at least 3 courses^3^	0.81 (0.75;0.87)	0.80 (0.74;0.87)	0.25 (0.18;0.31)

Mean duration of intervention (days)		210 (198;225)	

Participation in individual meetings and sessions was determined by the individual's needs and preferences, which were assessed by the counsellor and participant in collaboration. Target rates were thus not necessarily 1.00 and for that reason the conventional terminology of compliance is avoided.

^1^A network comprised one to four persons but is considered here as one unit. Except for the network meetings, networks were not invited to attend counselling or courses; some participated in selected sessions, however. ^2^Either the patient or the caregiver, or both, could register for calls.^3^Participation in three sessions of constructivist counselling (of which at least two were face-to-face, not telephone) or courses was considered a high degree of concordance, given the flexible nature of the intervention.

### Resource utilisation

Table 4 details the estimated resource utilisation per patient (ex capital costs) of the intervention. Constructivist counselling included five types of sessions: the first three were scheduled as individual sessions for patients and caregivers, respectively, while the last two were scheduled as combined sessions for patients, caregivers and networks. Overall, the constructivist counselling activity used almost 6 1/2 hours of counsellor time, 2 1/2 hours of patient time and 2 1/2 hours of caregiver time. Network time amounted to an average of 42 minutes due to low participation rates.

**Table 4:** Resource utilisation by health care personnel, patients, caregivers, and patient networks in a multifaceted intervention for mild Alzheimer's disease.

	Professionals' time^1^	Patients' time (n = 157)	Caregivers' time (n = 157)	Network's time^2^ (n = 157)
Constructivist counselling	379 (361;394)	199 (188;210)	202 (188;213)	42 (32;54)
Telephone counselling	78 (69;87)	12 (9;17)	48 (41;56)	Not applicable
Courses	541 (500;580)	472 (436;501)	465 (433;495)	148 (112;195)
Transportation	269 (256;285)	463 (440;486)	458 (433;480)	169 (121;239)

Total	1,267 (1,211;1,309)	1,146 (1,085;1,205)	1,172 (1,007;1,230)	358 (272;462)

^1^For counselling activities and transportation, the times given are those used by the counsellor only. Time used in courses is the sum of time used by the counsellor, volunteers and teachers. Professionals' times are not equal to row-sum of other parties' times as the ratio of professional to participant was not 1:1 and unsuccessful telephone calls added to counsellor time but not to any other party's time. ^2^A network comprised one to four persons but is considered here as one unit.

Values are mean minutes of time (95% confidence interval).

While all courses could accommodate up to 12 participants, for logistic reasons most courses were conducted with fewer participants. The variation in professional time used was thus due to variation in the number of participants (class size) rather than variation in course duration. Overall, the course activity used nine hours of professional time, associated with an average of almost eight hours of face-to-face time for patients and caregivers.

The resource utilisation associated with transportation reflects non-productive professional and participant time. The majority of counselling sessions (two-thirds) were held at health care institutions; hence the greatest transportation burden was on the patients and caregivers, who each spent an average of almost eight hours on transportation between their homes and the institution. Professionals had an average transportation burden of about four hours, which was attributable to the one-third of counselling sessions that were conducted in patients' own homes.

Altogether, the resource utilisation associated with the intervention amounted to 19-21 hours each for the professionals, patients and caregivers, and 6 hours for the network contacts.

### Intervention costs

The total cost of the intervention was 2,020 EUR (95% CI 1,929;2,106) per dyad. The distribution of costs was approximately 53% in the health care sector, 17% incurred by the patient, 21% incurred by the caregiver and 9% incurred by the patient's network. Total intervention costs thus demonstrated an approximate 1:1 cost sharing ratio between public and private pockets. Intervention costs are further detailed in Table 5.

**Table 5:** Intervention costs of multifaceted intervention for mild Alzheimer's disease.

		Indirect costs (time costs)		
		
	Direct costs^1^	Patient (n = 157)	Caregiver (n = 157)	Network (n = 157)
Constructivist counselling	307 (294;320)	41 (39;44)	55 (50;59)	59 (30;53)
Telephone counselling	63 (57;70)	3 (2;4)	11 (9;13)	Not applicable
Courses	449 (410;479)	98 (90;105)	128 (115;141)	44 (33;56)
Transportation^2^	251 (240;267)	205 (192;214)	233 (218;247)	73 (55;101)

Total	1,070 (1,029;1,109)	346 (325;364)	428 (397;459)	175 (148;208)

^1^Direct costs include time costs for counsellors plus an overhead of 20% to account for capital costs. ^2^Transportation costs include the direct taximeter or ticket cost plus the indirect costs of peoples' time.

Costs are in 2008-EUR (95% confidence interval).

### Sensitivity analysis

**Figure 1:**
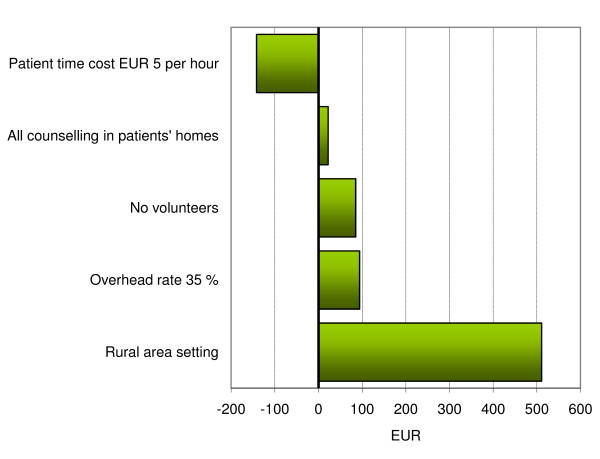
**Impact of alternative scenarios of the total intervention cost of a multifaceted intervention for patients with mild Alzheimer's disease and their primary caregivers (EUR 2,020 in base-case analysis)**.

**Figure 2:**
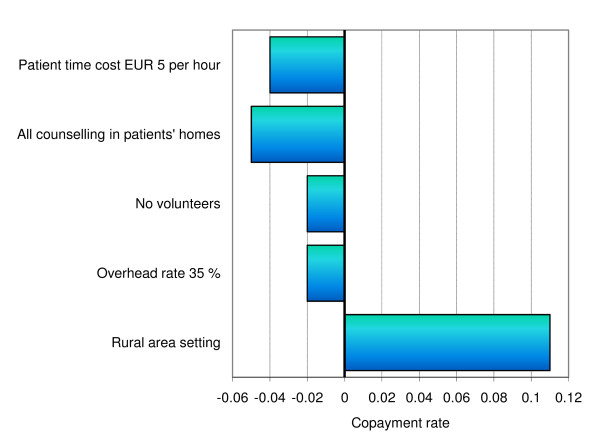
**Impact of alternative scenarios of the proportion of private copayment by patients with mild Alzheimer's disease and their primary caregivers attending a multifaceted support programme (0.47 in base-case analysis)**.

The study results are context-specific due to the intervention's resource utilisation being dependent on e.g. infrastructure, professional roles, participation rates and item cost differences between markets. Figure 1 presents the results of selected sensitivity analyses relating to the total intervention cost. Figure 2 presents sensitivity analyses relating to the copayment rate. First, there is a choice between providing counselling in the patients' homes or at health care institutions (the base-case was one-third of sessions in the patients' homes and the remainder at institutions). Had all counselling been conducted in the patients' homes, transportation costs would shift from participants to the health care sector, resulting in an overall cost increase of, on average, 22 EUR. The copayment rate would accordingly decrease by 0.05. A different issue relates to the use of volunteers, who assisted professionals in conducting patient courses, at a time cost lower than that of health care professionals. Had no volunteers been available, the intervention would have required more professional staff (e.g. nurses); the cost to health care providers would then increase by an average of 85 EUR per dyad, while the copayment rate would decrease by 0.02.

It can be argued that patient time should be valued using a wage rate less than that of fully productive individuals. The average impact of valuing patient time using a fixed rate of EUR 5 per hour was a total cost reduction of EUR 142 per dyad and a copayment rate reduction of 0.04. The average impact of increasing (or reducing) the overhead rate by 15% was an extra (or reduced) cost of EUR 95 per dyad, which would affect the copayment rate by -0.02 (+0.02). Finally, the impact of transportation costs was analysed as these may vary considerably across settings due to e.g. population density and degree of specialisation of health care institution. Had the average one-way distance (and time used on transportation) been doubled, average participant costs would increase by EUR 511 per dyad and the copayment rate would increase by 0.11.

## Discussion

This paper reports the economic consequences to both private and public funds of a best-bet intervention aimed at patients with mild AD and their primary caregivers. The intention of this work was to examine the extent to which long-standing intervention involving not only patients but also caregivers requires direct and indirect copayment, which ultimately may affect equity in access to health care. The main finding was that intervention in mild AD may come at a relatively low cost to public funds but, at the same time, it requires significant copayment from private parties, which can be summarised in a cost-sharing ratio between private and public pockets of almost 1:1.

The concurrent and detailed measurement of patient and caregiver time required for the successful intervention in mild AD could have been performed in other intervention studies, yet this seems to have been neglected in studies reported in the literature. This is in contrast to a strong economic theoretical recommendation to include patient and caregiver time as essential resource utilisations: costing from a societal perspective should include all resource utilisation irrespective of who invests it. The absence of such practice could be due to a lack of consensus regarding a valid and reliable framework for costing informal care [23-25] or to the more pragmatic issue of whether the required effort of measurement is worthwhile relative to the benefit.

From a theoretical point of view, a patient is 'occupied' when he or she is receiving treatment and thus benefits are sacrificed elsewhere i.e. the person cannot gain utility from leisure time or financial earnings (if in paid employment). The minimum value of benefits forgone is, using the opportunity cost approach, approximated by the individual's net (if leisure time is used) or gross wage (if time off from work is used) [26]. However, as most AD patients are not in paid work, a hypothetical wage rate (we used the age- and gender-matched national average) may not reflect the true value of patients' time. We conducted sensitivity analysis using a lower rate, in order to be flexible about using the average wage rate to value patient time. However, only a moderate impact on the overall cost-sharing ratio was demonstrated.

The term 'concordance' was used instead of the more conventional term 'compliance'. This was in consensus with recent criticism that 'compliance' denotes the extent to which the patient obeys the clinician rather than takes an active role in the doctor-patient relationship [27,28]. In this context, concordance refers to the extent to which the collaboration between counsellor and participant results in participation in individual components of the intervention. Relating to external validity, the high rates of participation achieved in the current trial were higher than expected and may be explained by the fact that participants seemed to be socioeconomically advantaged relative to the average citizen and, not least, that the trial was undertaken by highly dedicated professional staff. Lower rates of participation may be expected in routine practice, although that would not be expected to significantly influence the ratio of private to public costs.

There are some uncertain variables in relation to local infrastructure - both of the health care sector (e.g. whether relevant professionals are available at the nearest institution) and the private sector (e.g. what is the average distance from participants' homes to the institution). We observed significant variation across the five county districts of the trial, partly due to the free choice of participating centres to conduct counselling in the patients' homes or at health care institutions. Similarly, some centres were able to run highly efficient courses with all places filled, while others suffered low volume due to location in rural areas. Such variation affects not only total intervention costs but also shifts costs back and forth between private and public pockets.

Several issues are of note for community health planners who might consider adaptation of the intervention described here. First, the study population represents a relatively resourceful group as they are at an early disease stage and are socioeconomically advantaged. While the study is one of the few to describe an intervention targeted to this population, the results are not likely to be generalisable to more severe stages of AD. Second, favourable resource utilisation and costs are irrelevant if there is no evidence for a clinical effect and the most useful approach would be a synthesis of information as a cost-effectiveness evaluation. These analyses are currently in progress. A third remark relates to budget impact, which should be estimated by multiplying not only the *n *of a target population with the intervention cost, but should also take into account that a certain proportion of eligible dyads reject the invitation to participate and a further proportion drop out after initially agreeing to participate. We have no estimates on the former proportion, whereas the latter appeared to be 0.12. Consideration should also be given to recruitment of participants, that is, whether existing regimens can accommodate recruitment or whether extra resources are required to establish a regimen for screening and inviting candidate participants. Such costs are not included in the present analysis. Finally, start-up costs of e.g. recruiting and educating staff, developing informational packages etc. should be expected (these were not included in the current study).

It has long been argued that disparities in the use and quality of care are particularly evident in long-term care, but the first attempt to summarise this evidence was reported only recently [29]. Much of the evidence arises from the USA and focuses on racial disparities, where Blacks generally appear to have a lower health care use than Hispanics. One study, however, shows that individuals with low socioeconomic status generally underuse health care; explanatory variables appear to include organisational and geographic characteristics of care provision  [30]. This could be synonymous with a hypothesis that requirements of considerable participant time input and high transportation costs pose a restriction to health care access. However, the direct question of whether a 1:1 cost-sharing ratio between private and public finances restricts access to care for some groups relative to others remains unanswered.

## Conclusion

Intervention in mild Alzheimer's disease can be undertaken at a relatively low cost to public funds. However, policy planners should pay attention to the significant private costs associated with an intervention, which may ultimately pose a threat to equity in access to health care.

## Competing interests

The DAISY study was supported by the National Board of Social Services at the Danish Ministry of Social Affairs, the Danish Ministry of Health, the Danish Health Foundation and the Research Council of the Copenhagen Hospital Cooperation. The authors declare that they have no competing interests.

## Authors' contributions

RS designed the cost analysis, undertook all statistical analyses and drafted the manuscript. JS participated in conceiving the economic perspective of the DAISY study, from which participants in the present work was sampled, and in editing this manuscript. FW, AE, DB and GW conceived and led the DAISY study and assisted in editing this manuscript. All authors read and approved the final manuscript.

## Pre-publication history

The pre-publication history for this paper can be accessed here:

http://www.biomedcentral.com/1472-6963/9/215/prepub
